# Development of a fluorescent ASFV strain that retains the ability to cause disease in swine

**DOI:** 10.1038/srep46747

**Published:** 2017-04-24

**Authors:** Manuel V. Borca, Vivian O’Donnell, Lauren G. Holinka, Brent Sanford, Paul A. Azzinaro, Guillermo R. Risatti, Douglas P. Gladue

**Affiliations:** 1 Agricultural Research Service Plum Island Animal Disease Center, Greenport, NY 11944, US; 2 Department of Homeland Security, Plum Island Animal Disease Center, Greenport, NY 11944, USA; 3 Departments of Pathobiology and Veterinary Science, University of Connecticut, Storrs, CT 06269, USA; 4 Oak Ridge Institute for Science and Education (ORISE), Oak Ridge, TN 37831, USA

## Abstract

African swine fever is a contagious and often lethal disease for domestic pigs with a significant economic impact for the swine industry. The etiological agent, African swine fever virus (ASFV), is a highly structurally complex double stranded DNA virus. No effective vaccines or antiviral treatment are currently commercially available. We present here the development of a strain of ASFV that has been shown to retain its ability to cause disease in swine, efficiently replicate in swine macrophage and that is fluorescently tagged. The insertion of an EGFP cassette replacing the reading frames for two neighboring genes, MGF360-13L and MGF360-14L, in highly virulent field isolate Georgia/2007, did not affect virus replication in cell cultures and did not affect disease progression in swine, the natural host for ASFV. A virulent fluorescently tagged ASFV is a suitable tool to conduct pathogenesis studies in swine, study on virus-macrophage interaction and to run large scale screens that require a sensitive high throughput output. Utilizing an EGFP reporter system for observing ASFV replication and infectivity can circumvent the time and labor-intensive steps associated with viral antigen-based assays such as the observation of hemadsorption or cytopathic effect.

African swine fever virus (ASFV) is the only member of the Asfarviridae family and is the etiological agent of African swine fever (ASF). ASFV has large and complex genomic structure consisting of a linear double stranded DNA that contains approximately 180–190 kilobase pairs with more than 150 ORFs. ASF causes a spectrum of disease, from highly lethal to sub-clinical, depending on host characteristics and the virus strain^1^. ASF is endemic in several sub-Saharan African countries. In Europe, the disease is endemic in Sardinia (Italy) and outbreaks have been recorded in the Caucasus region since 2007, affecting Georgia, Armenia, Azerbaijan and Russia and more recently in Ukraine, Belarus, Lithuania, Latvia and Poland, threatening to disseminate into neighbouring West European countries^2^. The contagious and often lethal nature of ASFV in domestic pigs has substantial economic consequences for the swine industry in countries where infection occurs^3^.

Currently there is no commercial vaccine available for ASF and outbreaks are controlled by animal quarantine and elimination of affected animals^4^,^5^. In addition, there are no antivirals that are available to control ASF in swine. A few antiviral agents have been discovered, but only tested in cell culture using non-virulent cell culture adapted strains^5^,^6^,^7^. Identification of new and novel antiviral agents would require a system that allows for high throughput detection of virus replication using a virulent virus strain, in a system that mimics the host.

Previous work in adaptation of ASFV strains to cell cultures has resulted in large deletions occurring in ASFV in both the left and right variable regions^8^,^9^,^10^,^11^. These large deletions typically affect several distinct multigene families (MGF), characterized as genes that contain partially repetitive sequence similarities. The different MGF groups were named to reflect the average lengths of the predicted gene product^12^,^13^. Importantly, this adaptation to cell cultures has coincided with the decreased ability of ASFV to grow in swine macrophages, and the attenuation of ASFV in swine. Therefore, these adapted viruses may not closely represent antigenically and/or pathogenically to those causing swine disease in the field^9^,^14^.

In a previous study, several smaller deletions in the MGF family region were tested for their role in *Ornithodoros porcinus* ticks, the arthropod host for ASFV^15^, in this study one of these smaller mutants deleted area in the left variable region of ASFV comprising of only MGF360-12L to MGF360-14L. This small deletion had no effect on growth in swine macrophages, but a defect in growth in ticks. This study suggested that these genes are not required for replication in swine, but only in ticks. We hypothesized that this site could be used as a safe harbor site to express a foreign protein of interest without affecting virus replication in swine macrophages or virus virulence in swine.

To date there is no available virulent ASFV strain that can be used for hi-throughput assays. Monitoring infection relies on time consuming and labor intensive viral antigen-based assays as hemadsorption (HA) or cytopathic effect detection. Quantification of viral infection on a large scale is also unfeasible, and requires laborious counting and subjectivity. In order to use an ASFV strain for hi-throughput screening the strain would require that several conditions be met. First, the strain would require some sort of reporter that could be quantitated mechanically, such as green fluorescent protein (GFP). Second, the strain would have to grow in macrophage cultures, as growth in macrophage cultures has in the past been a partial predictor for the ability of ASFV to grow *in vivo*. Finally, a strain would have to retain its ability to be virulent in swine. The introduction of a fluorescent tag attached to ASFV structural protein p54 has been previously described using a non-virulent cell culture adapted strain^16^ and using 5-bromo-2′-deoxyuridine selection to replace the ASFV thymidine kinase (TK) locus with GFP in field isolates^17^, however in both cases the resulting virus were not shown to be virulent in swine, or to still be able to efficiently replicate in swine macrophages.

Here we report the development of a genetically modified ASFV strain that contains an enhanced green fluorescent protein (EGFP) as a reporter gene. Using the highly virulent parental virus ASFV Georgia/2007 (ASFV-G), we developed a novel virus strain, ASFV-G-ΔMGF13/14-EGFP, which has deleted MGF360-13L and MGF360-14L genes and replaced with EGFP under the control of a p72 promoter. ASFV-G-ΔMGF13/14-EGFP maintains the ability to grow in macrophages and the ability to cause full disease progression in swine, with similar disease characteristics to the parental virus. This EGFP reporter virus could be used for both rapid, hi-throughput *in vitro* screenings as well as in pathogenesis studies in swine.

## Results and Discussion

### Introduction of EGFP in the MGF360-13/14 locus

**Figure 1:**
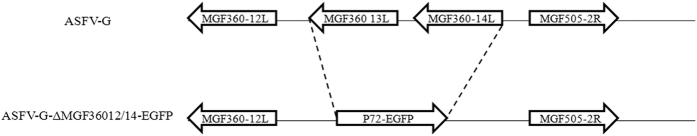
Schematic representation of the MGF360-13L and MGF360-14L gene regions deleted in ASFV-G-ΔMGF36013/14 and replaced with the p72EGFP reporter gene cassette.

Recombinant ASFV-G-ΔMGF-13/14-EGFP was generated by homologous recombination between the parental ASFV-G genome and a recombination transfer vector following infection and transfection of swine macrophage cell cultures^18^. The recombinant transfer vector (p72ΔMGF13/14-EGFP) (Fig. 1) contained flanking genomic regions, the left arm is located between genomic positions 29352-30551 and the right arm is located between genomic positions 32914-33615. A reporter gene cassette containing the green fluorescent protein (EGFP) gene with the ASFV p72 late gene promoter was inserted between these arms. This construction created a deletion of the MGF360-13L and MGF360-14L genes, with an insertion of EGFP in the same area (Fig. 1). Recombinant transfer vector p72ΔMGF13/14-EGFP was obtained by DNA synthesis (Epoch Life Sciences, Sugar Land, TX, USA). Macrophage cell cultures were infected with ASFV-G and transfected with p72ΔMGF13/14-EGFP. Recombinant viruses representing independent primary foci were purified to homogeneity by successive rounds of limiting dilution purification.

### Analysis of the ASFV-G-ΔMGF13/14-EGFP genome sequence relative to parental ASFV-G genome sequence

To evaluate the accuracy of the genetic modification and the integrity of the genome of the recombinant virus, full genome sequences of ASFV-G-ΔMGF13/14-EGFP and parental ASFV-G were obtained using NGS on the Ion Torrent PGM and compared. First, a full-length genome comparison between parental ASFV-G and ASFV Georgia 2007/1^19^ was performed. The following differences were observed between these two viruses (nucleotide positions are provided based on ASFV Georgia 2007/1 GenBank accession no. FR682468): (i) two nucleotide insertions, T at position 433 and A at position 441, in a non-coding segment of the genome; (ii) two nucleotide deletions, T at position 1602 and T at position 1603, in the MGF360-1L gene ORF resulting in a frame shift; (iii) a nucleotide insertion, T at position 1620, in the MGF360-1L gene ORF resulting in a frame shift; a nucleotide mutation of A to G at position 97321 resulting in a silent mutation in ORF B438L; (v) a nucleotide mutation of C to G at position 166192 resulting in a residue substitution (Ala to Pro) at residue position 85 in ORF E199L; and (vi) a nucleotide insertion of T at position 183303, a non-coding segment of the genome. Second, a full-length genome comparison between ASFV-G-ΔMGF13/14-EGFP and parental ASFV-G was performed. The DNA sequence of ASFV-G-ΔMGF13/14-EGFP revealed a deletion of 2,363 nucleotides in ORFs MGF360-13L and MGF360-14L relative to parental ASFV-G that corresponds with the introduced modification.

The consensus sequence of the ASFV-G-ΔMGF13/14-EGFP genome showed an insertion of 1300 nucleotides replacing ORFs MGF360-13L and MGF360-14L corresponding to the p72EGFP cassette sequence introduced. Besides the insertion of the p72EGFP cassette, no additional changes were observed. In summary, ASFV-G-ΔMGF13/14-EGFP virus did not accumulate any substantial mutations during the process of homologous recombination and consequent plaque purification steps.

### EGFP expression in ASFV-G-ΔMGF13/14-EGFP

**Figure 2:**
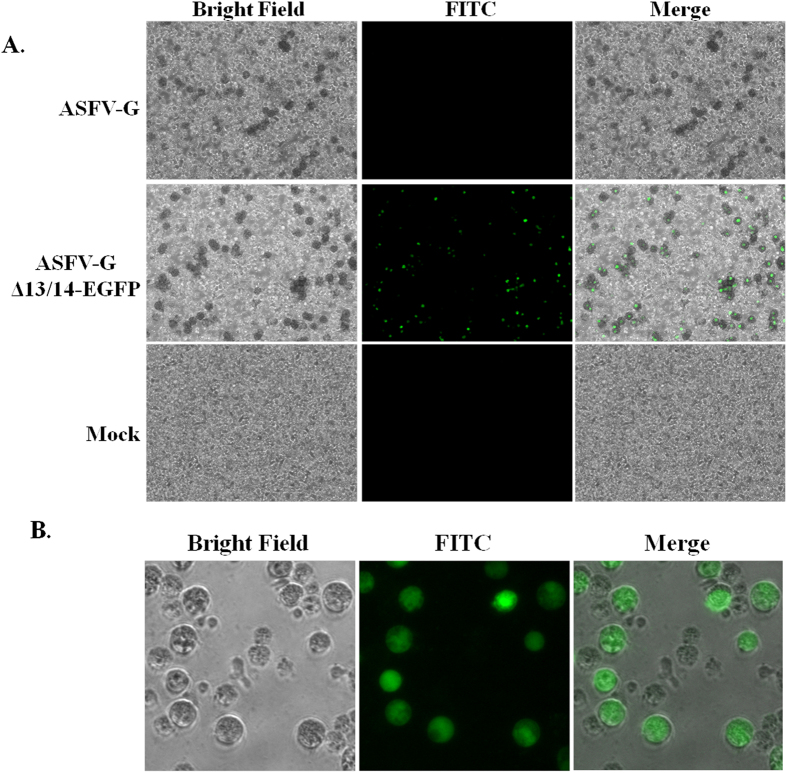
(**A**) Primary swine macrophages were inoculated with the indicated virus at an MOI of 0.5 or mock infected, in the presence of red blood cells. In the first column bright field images were taken 20hpi, and observed for hemadsorption. In the second column fluorescence was examined using a FITC filter. The two images were merged in the third column. (**B**) Swine macrophages were inoculated with ASFV-G Δ13/14 EGFP and examined at 16hpi using bright field images in the first column and in the second column fluorescence was examined using a FITC filter.

Expression of EGFP in the recombinant virus during infection was tested using fluorescent microscopy in cells infected with ASFV-G-ΔMGF13/14-EGFP or parental virus ASFV-G. Cells were infected with a MOI of 0.5 or mock infected, in 6-well plates with the addition of red blood cells and observed at 16 (hpi) and examined by fluorescent microscopy. EGFP expression was clearly observed by fluorescence in macrophages displaying hemadsorption (HA) at 16 hpi (Fig. 2). Importantly, no observed EGFP activity was detected in cells infected with ASFV-G.

Furthermore, it was assessed if the deletion of ΔMGF13/14 and the expression of EGFP genes may alter in any way the ability of ASFV-G-ΔMGF13/14-EGFP infected cells to hemadsorb, a classic indicator of ASFV infectivity. Cells were infected with ASFV-G-ΔMGF13/14-EGFP or parental virus ASFV-G with the addition of red blood cells, and sequentially observed during 24hpi detecting the presence of HA as well as fluorescence. In ASFV-G-ΔMGF13/14-EGFP infected cells all hemadsorbing positive cells expressed EGFP (Fig. 2A). As expected, in ASFV-G infected cells, none of hemadsorbing positive cells had any detectable fluorescent activity. Therefore, expression of EGFP does not affect the phenomenon of HA. Furthermore, EGFP activity is easily detected by fluorescence in macrophages infected with ASFV-G-ΔMGF13/14-EGFP and the genetic modifications involved in the development of this strain do not alter the ability of the infected cell to mediate HA. The cellular localization of EGFP is widespread across the entire cytoplasm of the cell during late time points during infection (Fig. 2B). As expected there was no EGFP signal during early gene expression time points before 8hrs (data not shown).

### Ability of ΔMGF13/14-EGFP to grow *in vitro*

**Figure 3:**
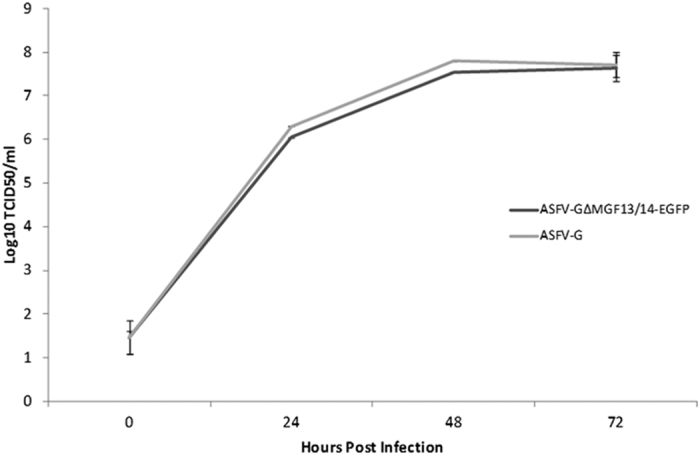
*In vitro* growth kinetics of the in ASFV-GΔMGF13/14-EGFP and parental ASFV-G viruses. Primary swine macrophage cell cultures were infected MOI of 0.1 with either ASFV-GΔMGF13/14-EGFP or parental ASFV-G virus. Virus yields were estimated at the indicated times post infection by titration in primary swine macrophage cell cultures. Data represent means and standard deviations from two independent experiments. The sensitivity of virus detection was ≥1.8 HAD_50_/ml.

Field ASFV isolates fully grown in peripheral blood derived primary swine macrophages, the primary cell targeted by ASFV during infection in swine, but not in otherstable cell cultures To evaluate if deletion of ΔMGF13/14 genes and the expression of EGFP genes may alter the ability of ASFV-G-ΔMGF13/14-EGFP to replicate in swine macrophage its kinetics of replication was assessed and compared relative to parental ASFV-G in a multistep growth curve (Fig. 3). Cell cultures were infected at an MOI of 0.1, supernatant samples were collected at 2, 24, 48, and 72 h post infection (hpi) and virus yields titrated also in swine macrophage cell cultures. Results demonstrated that ASFV-G-ΔMGF13/14-EGFP displayed almost identical growth kinetics as parental ASFV-G virus, therefore suggesting that genomic changes in ASFV-G-ΔMGF13/14-EGFP does not affect growth *in vitro*.

### Comparison of virus detection sensitivity using direct fluorescence versus hemadsorption (HA)

**Figure 4:**
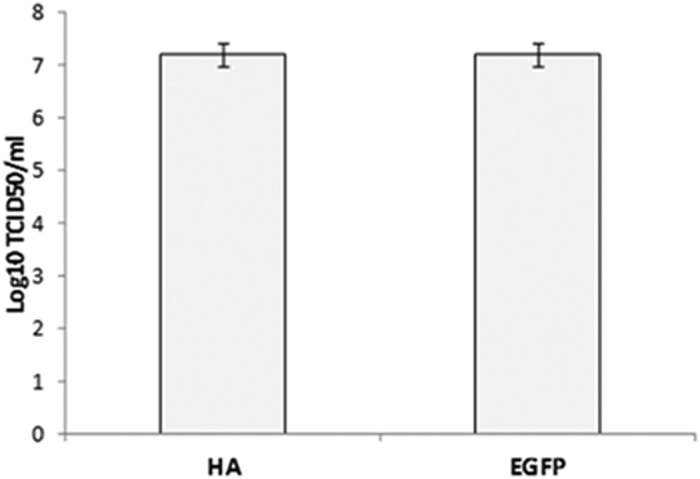
Comparison of the sensitivity of viral titers by determining positivity of wells either by hemadsorption or by detection of EGFP of the ASFV-GΔMGF13/14-EGFP viral stock. Values are expressed as log_10_ HAD_50_/ml or log_10_ TCID_50_/ml. The sensitivity of virus detection was ≥log_10_ 1.8 HAD_50_/ml.

The sensitivity of using EGFP as a detection system was tested against the use of conventional HA, a detection system proven to be more sensitive than real time-RT-PCR^20^. Presence of ASFV-G-ΔMGF13/14-EGFP in ten-fold dilution samples was simultaneously detected by fluorescence and HA. ASFV-G-ΔMGF13/14-EGFP dilutions were seeded on primary swine macrophage cell cultures in 96-well plates, after three days the presence of virus was assessed either by HA to calculate HAD_50_ or by visualizing EGFP activity by fluorescence microscopy to calculate the TCID_50_. Remarkably, results demonstrated identical titer values using both detection methodologies (Fig. 4).

### Assessment of ASFV-G-ΔMGF13/14-EGFP virulence in swine

Parental ASFV-G, a highly virulent isolate, causes disease and death in 100% of infected swine when intramuscularly (IM) inoculated at a dose of 10^2^ HAD_50_. To determine if genetic modifications introduced in ASFV-G-ΔMGF13/14-EGFP may alter its virulence we compared the outcome of infection in swine inoculated with ASFV-G-ΔMGF13/14-EGFP along to animals infected with parental ASFV-G. Eighty to ninety-pound pigs were IM inoculated with 10^2^ HAD_50_ of either ASFV-G or ASFV-G-ΔMGF13/14-EGFP. In both groups of animals an increased body temperature (>104^o^F) was observed at four days post infection (Table 1). Pigs presented clinical signs associated with the disease, including anorexia, depression, purple skin discoloration, staggering gait, and diarrhea. Clinical signs progressed over time, and animals either died or were euthanized *in extremis* by day 7 post infection. The progression of ASFV observed in animals infected with ASFV-G-ΔMGF13/14-EGFP were similar to those seen in the parental virus inoculated animals (Table 1).

**Figure 5:**
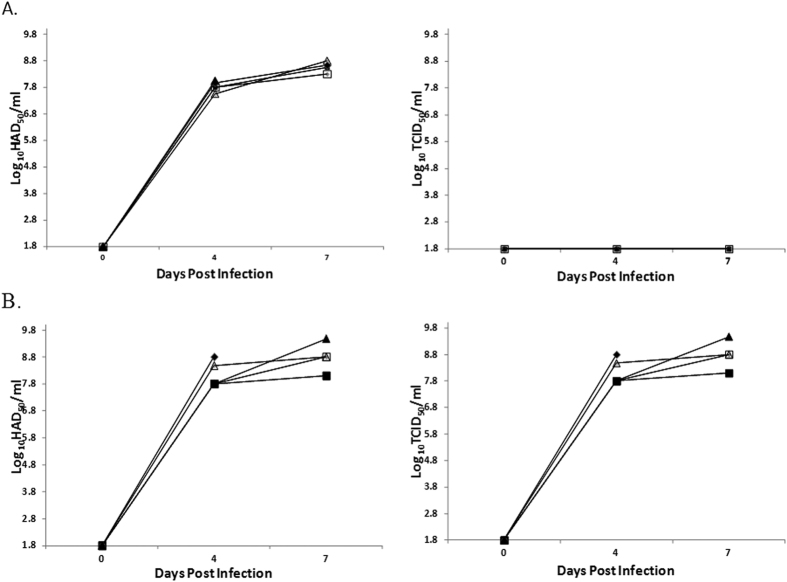
Virus titers in blood samples obtained from pigs that were infected with 10^2^ HAD_50_ of either (**A**) ASFV-G or (**B**) ASFV-G ΔMGF13/14-EGFP. Panels on the left show titers calculated by HA while panels on the right using fluorescent microscopy. Each symbol represents a different animal. Values are expressed as log_10_ HAD_50_/ml or log_10_ TCID_50_/ml. The sensitivity of virus detection was ≥log_10_ 1.8 HAD_50_/ml.

Viremia in experimentally inoculated animals was quantified at different days post-infection. All animals inoculated with 10^2^ HAD_50_ of ASFV-G-ΔMGF13/14-EGFP or parental ASFV-G had very high virus titers in blood until the day of their death, therefore the progression of the viremia in ASFV-G-ΔMGF13/14-EGFP (Fig. 5A) was similar to that of swine inoculated with ASFV-G (Fig. 5B). Therefore, it is clear that genetic modification involving the deletion of MGF36013L and MGF36014L or the inclusion of EGFP gene do not meaningfully alter the virulence of ASFV-G-ΔMGF13/14-EGFP in pigs. Animals infected with ASFV-G-ΔMGF13/14-EGFP develop clinical disease kinetics indistinguishable from those observed in animals infected with parental ASFV-G. Similarly, viremia levels in ASFV-G-ΔMGF13/14-EGFP infected animals overlap those observed in animals infected with ASFV-G. Importantly, blood samples obtained from animals infected with ASFV-G-ΔMGF13/14-EGFP were able to be titrated using fluorescence, giving the same TCID_50_ values as HAD_50_, suggesting stability of the EGFP cassette after the virus has undergone an *in vivo* infection. Therefore, using EGFP as a reporter gene assessed by fluorescent microscopy is as effective as HA as a methodology of detecting presence of ASFV-G-ΔMGF13/14-EGFP-infected cells in primary swine macrophages cultures. Although we did observe more EGFP expressing cells than HA positive cells especially in low titration dilutions, there were no differences in calculated titers. It is possible that fluorescent detection is slightly more sensitive than HA as it can be observed as soon as the EGFP protein is produced rather than requiring enough protein expression present to mediate the presence of hemadsorption.

**Figure 6:**
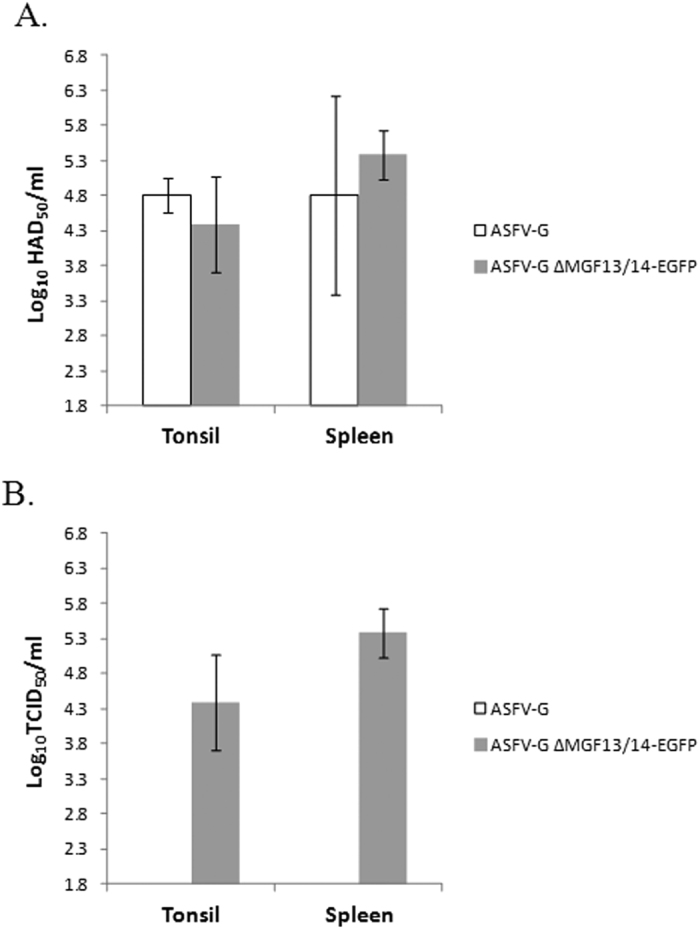
Virus titers in tissue samples obtained from pigs that were infected with 10^2^ HAD_50_ of either ASFV-G or ASFV-G ΔMGF13/14-EGFP. (**A**) Titers were calculated by HA (**B**) Titers were calculated using fluorescent microscopy. Values are expressed as log_10_ HAD_50_/ml or log_10_ TCID_50_/ml. The sensitivity of virus detection was ≥log_10_ 1.8 HAD_50_/ml.

To further test the stability of the EGFP protein insertion we isolated virus from tonsils and spleen samples obtained from the same animals. We found comparable virus titers in tonsils and spleen of animals infected with either virus. In addition, ASFV-G-ΔMGF13/14-EGFP titer values in both tissues were identical regardless of whether or not they were calculated by fluorescence microscopy of HA (Fig. 6).

As a summary, we described here for the first time the development of a highly virulent ASFV strain expressing EGFP as reporter gene. We show that insertion of EGFP in the MGF360-13/14 locus had no effect on virus growth in swine macrophages cultures or virulence in swine. Having an EGFP reporter virus will allow the ability to adapt hi-throughput methods to ASFV that could be used for screening antiviral compounds or for other large scale genomic based screens to identify cellular factors involved in ASFV replication. Having the ability to conduct screens in swine macrophages, the natural target cell during animal infection, with a virus that is highly infectious that may have the ability to mimic a naturally occurring infection in cell cultures. This is of great importance, as ASFV-G-ΔMGF13/14-EGFP has not been adapted to cell culture, still is able to cause disease in swine and has to the ability to grow in swine macrophage cultures, indicates that it is likely that ASFV-G-ΔMGF13/14-EGFP utilizes the same cellular environment or cellular pathways that occur in virulent strains of ASFV.

## Materials and Methods

### Cell cultures and viruses

Primary swine macrophage cell cultures were prepared from defibrinated swine blood as previously described by Zsak *et al*.^18^. Briefly, heparin-treated swine blood was incubated at 37 °C for 1 hour to allow sedimentation of the erythrocyte fraction. Mononuclear leukocytes were separated by flotation over a Ficoll-Paque (Pharmacia, Piscataway, N.J.) density gradient (specific gravity, 1.079). The monocyte/macrophage cell fraction was cultured in plastic Primaria (Falcon; Becton Dickinson Labware, Franklin Lakes, N.J.) tissue culture flasks containing macrophage media, composed of RPMI 1640 Medium (Life Technologies, Grand Island, NY) with 30% L929 supernatant and 20% fetal bovine serum (HI-FBS, Thermo Scientific, Waltham, MA) for 48 hours at 37 °C under 5% CO_2_. Adherent cells were detached from the plastic by using 10 mM EDTA in phosphate buffered saline (PBS) and were then reseeded into Primaria T25, 6- or 96-well dishes at a density of 5x10^6^ cells per ml for use in assays 24 hours later.

ASFV Georgia (ASFV-G) was a field isolate kindly provided by Dr. Nino Vepkhvadze, from the Laboratory of the Ministry of Agriculture^1^ in Tbilisi, Republic of Georgia^9^.

Comparative growth curves between ASFV-G, ASFV-GΔMGF13/14-EGFP were performed in primary swine macrophage cell cultures. Preformed monolayers were prepared in 24-well plates and infected at a MOI of 0.1 (based on HAD_50_ previously determined in primary swine macrophage cell cultures). After 1 hour of adsorption at 37 °C under 5% CO_2_ the inoculums were removed and the cells were rinsed two times with PBS. The monolayers were then rinsed with macrophage media and incubated for 2, 24, 48, 72 and 96 hours at 37 °C under 5% CO_2_. At appropriate times post-infection, the cells were frozen at ≤−70 °C and the thawed lysates were used to determine titers by HAD_50_/ml in primary swine macrophage cell cultures. All samples were run simultaneously to avoid inter-assay variability. For immunofluorescence studies, 2 × 10^6^ cells were seeded in 24 well plates on glass coverslips, after the indicated time, coverslips were fixed with 4% paraformaldehyde for 15 min, and washed twice in PBS.

Virus titration was performed on primary swine macrophage cell cultures in 96-well plates. Virus dilutions and cultures were performed using macrophage medium. Presence of virus was assessed either by HA or by fluorescent microscopy three days after being plated, virus titers were calculated by the Reed and Muench method^21^ and expressed as HAD_50_ or TCID_50_ as detected by the presence HA or fluorescence.

### Next Generation sequencing of ASFV genomes

ASFV DNA was extracted from infected cells and quantified as described earlier^9^. Full-length sequencing of the virus genome was performed as described elsewhere^9^. Briefly, one microgram of virus DNA was enzymatically sheared and the resulting fragmented DNA size distribution was assessed. Adapters and library barcodes were ligated to the fragmented DNA. The appropriate size range of the adapter-ligated library was collected using the Pippin Prep™ system (Sage Science, Beverly, MA) followed by normalization of library concentration. The DNA library was then clonally amplified onto ISPs and enriched. Enriched template ISPs were prepared and loaded onto Ion chips for sequencing using an Ion Torrent PGM™ instrument. Sequence analysis was performed using Galaxy (https://usegalaxy.org/) and CLC Genomics Workbench (CLCBio, Waltham, MA).

### Animal experiments

Animal experiments were performed under biosafety level 3 conditions in the animal facilities at PIADC where all methods were performed in accordance with the relevant guidelines and regulations following a protocol approved by the Plum Island Animal Disease Center Institutional Animal Care and Use Committee.

ASFV-GΔMGF13/14-EGFP was assessed for its virulence phenotype relative to the parental ASFV-G virus using 80–90 pound commercial breed swine. Five pigs were inoculated intramuscularly (IM) either with 10^2^ or 10^3^ HAD_50_ of ASFV-GΔMGF13/14-EGFP or 10^2^ HAD_50_ of ASFV-G. Clinical signs (anorexia, depression, fever, purple skin discoloration, staggering gait, diarrhea and cough) and changes in body temperature were recorded daily throughout the experiment.

## Additional Information

**How to cite this article:** Borca, M. V. *et al*. Development of a fluorescent ASFV strain that retains the ability to cause disease in swine. *Sci. Rep.* **7**, 46747; doi: 10.1038/srep46747 (2017).

**Publisher's note:** Springer Nature remains neutral with regard to jurisdictional claims in published maps and institutional affiliations.

## Figures and Tables

**Table 1:** Swine survival and fever response in ASFV-MGFΔ13-14 and parental virulent ASFV-G.

Virus	No. of survivors/total	Mean time to death (±SD)	Fever		
			Days to onset (±SD)	Duration Days (±SD)	Maximum daily temp, °F (±SD)
ASFV-G-MGFΔ13-14	0/5	7.2 (0.45)	4 (0.0)	3.2 (0.45)	105.32 (0.16)
ASFV-G	0/5	6.8 (0.55)	3.8 (0.45)	2.8 (0.84)	105.7 (0.55)
